# Phase I safety, efficacy, and biomarker response evaluations of three oral PD-L1 inhibitors: INCB086550, INCB099280, and INCB099318

**DOI:** 10.1136/jitc-2026-015334

**Published:** 2026-08-11

**Authors:** Hans Prenen, Sylvie Rottey, David J Pinato, Thierry Lesimple, Eric Van Cutsem, Marie Robert, Rachel Galot, Sarina A Piha-Paul, Pascale Tomasini, Nuria Kotecki, Rebecca Kristeleit, Christophe Le Tourneau, Ruth Plummer, Udai Banerji, Tarek Meniawy, Jason Howe, Jeannie Daniel, Jennifer Pulini, Susan Spitz, Xiaohua Gong, Molly Halloran, Antoine Italiano

**Affiliations:** 1 Center for Oncologic Research (CORE), University of Antwerp, Antwerp, Belgium; 2 Department of Oncology, University Hospital Antwerp, Edegem, Belgium; 3 Department of Medical Oncology and Drug Research Unit, Ghent University Hospital, Ghent University, Ghent, Belgium; 4 Department of Surgery and Cancer, Imperial College London, Hammersmith Hospital, London, UK; 5 Division of Oncology, Department of Translational Medicine, University of Piemonte Orientale, Novara, Italy; 6 Medical Oncology and Clinical Research Department, Eugène Marquis Centre, Rennes, France; 7 Digestive Oncology, University Hospitals Leuven and KU Leuven, Leuven, Belgium; 8 Medical Oncology, Institut de Cancérologie de l’Ouest, St. Herblain, France; 9 ARPEGO Network, St. Herblain, France; 10 Department of Medical Oncology, Institut Roi Albert II, Cliniques Universitaires Saint-Luc, Bruxelles, Belgium; 11 Department of Investigational Cancer Therapeutics, University of Texas MD Anderson Cancer Center, Houston, Texas, USA; 12 Early Phase Trials in Oncology Department, CNRS, INSERM, CRCM, APHM, Aix Marseille University, Marseille, France; 13 Medical Oncology, Jules Bordet Institute, Anderlecht, Belgium; 14 Department of Oncology, Guy’s and St Thomas’ NHS Foundation Trust and King’s College London, London, UK; 15 Department of Drug Development and Innovation (D3i), Institut Curie, Paris-Saclay University, Paris, France; 16 Sir Bobby Robson Cancer Trials Research Centre, Newcastle University and Newcastle Hospitals NHS Foundation Trust, Newcastle upon Tyne, London, UK; 17 Drug Development Unit, The Institute of Cancer Research and The Royal Marsden NHS Foundation Trust, London, UK; 18 Department of Oncology, St John of God Subiaco Hospital, Subiaco, Western Australia, Australia; 19 Research and Development, Incyte Corporation, Wilmington, Delaware, USA; 20 Department of Medicine, Institut Bergonié, Bordeaux, France; 21 Faculty of Medicine, University of Bordeaux, Bordeaux, France

**Keywords:** Pharmacodynamics - PD, Pharmacokinetics - PK, Solid tumor, Immune Checkpoint Inhibitor

## Abstract

**Background:**

Orally administered small-molecule programmed death ligand 1 (PD-L1) inhibitors may have the potential to improve patient outcomes in the treatment of a range of cancers compared with their antibody-based counterparts. A small molecule might achieve better tumor tissue penetration, and oral administration could significantly improve convenience and access for patients.

**Methods:**

Three phase 1 open-label, non-randomized, dose escalation, and expansion studies evaluated the safety, preliminary efficacy, pharmacokinetics (PK), and pharmacodynamics (PD) of three agents in patients with advanced solid tumors: INCB086550 (NCT03762447), INCB099280 (NCT04242199), and INCB099318 (NCT04272034).

**Results:**

Overall, 138, 182, and 104 patients received INCB086550, INCB099280, and INCB099318, respectively. Most had previously received ≥2 lines of cancer therapy for advanced or metastatic disease; 9.6%–16.5% had received prior immunotherapy. All three agents were rapidly absorbed and showed stable dose-dependent PK. With INCB086550, 88 patients (63.8%) had ≥1 treatment-related treatment-emergent adverse event (TEAE), and 19 (13.8%) had ≥1 treatment-related grade ≥3 TEAE. In total, 14 patients (10.1%) had a nervous system-associated TEAE for which an immune-mediated etiology could not be ruled out; events were predominantly peripheral sensory and motor neuropathies. With INCB099280 and INCB099318, 144 (79.1%) and 69 (66.3%) of patients had ≥1 treatment-related TEAE, and 25 (13.7%) and 12 (11.5%) had ≥1 treatment-related grade ≥3 TEAE, respectively. The most frequent immune-related adverse events were skin reactions (INCB099280 and INCB099318) and hepatitis (INCB099280). No dose-limiting toxicities (DLTs) occurred during dose escalation with INCB086550 or INCB099318; two DLTs occurred in two patients with INCB099280 (grade 2 vomiting with 600 mg once daily and grade 2 maculopapular rash with 800 mg two times per day). Overall objective response rates for INCB086550, INCB099280, and INCB099318 were 10.9% (95% CI 6.2% to 17.3%; n=15), 8.8% (95% CI 5.1% to 13.9%; n=16), and 8.7% (95% CI 4.0% to 15.8%; n=9), respectively. Target engagement and PD activity were demonstrated, including PD-L1 binding, and increases in cytokine and chemokine production, as well as T-cell activation and proliferation.

**Conclusions:**

Both INCB099280 and INCB099318 had an acceptable safety profile, with preliminary evidence of antitumor activity. The risk of immune-mediated neuropathy led to discontinuation of the clinical program for INCB086550.

**WHAT IS ALREADY KNOWN ON THIS TOPIC**

- Monoclonal antibody-based programmed death ligand 1 (PD-L1) pathway inhibitors have become part of standard of care in a range of advanced cancers.

**WHAT THIS STUDY ADDS**

- Three orally administered small-molecule PD-L1 inhibitors—INCB086550, INCB099280, and INCB099318—evaluated in phase 1 studies in patients with advanced solid tumors. INCB086550 was the first compound tested clinically, but immune-related adverse events of neuropathy led to development being discontinued. INCB099280 and INCB099318 had acceptable safety profiles and preliminary evidence of antitumor activity, but clinical development was discontinued for strategic reasons.

**HOW THIS STUDY MIGHT AFFECT RESEARCH, PRACTICE OR POLICY**

- The findings from these three studies demonstrate the feasibility of orally administered PD-L1 inhibitors as cancer therapies. There is a need to better understand the mechanistic differences between small molecules and monoclonal antibodies targeting this pathway for future compound development.

## Background

 Many cancers can evade or suppress the body’s natural immune responses, contributing to tumor growth.^1^ Blocking the programmed death ligand 1 (PD-L1) pathway in cancer cells can upregulate T-cell responses, stimulating production of specific cytokines such as interferon gamma (IFNγ), increasing proliferation and tumor infiltration by reactive CD8^+^ T cells, and inducing cytolytic activity against tumor cells.^2–4^ Monoclonal antibodies (mAbs) that block the PD-L1 pathway have shown improved response rates, duration of response (DOR), and/or overall survival compared with chemotherapy in the treatment of a range of advanced cancers.^5^ However, only a small fraction of patients responds to these therapies and there is a need for PD-L1 inhibitors that can be effective in a broader patient population.^6^ Additionally, there exists a need for agents with greater ease of administration that can be readily employed in combination regimens, which may improve patient outcomes and overcome drug resistance.^7 8^

Incyte Corporation (Wilmington, Delaware, USA) therefore developed three orally administered small-molecule PD-L1 inhibitors, INCB086550,^9^ INCB099280,^10^ and INCB099318,^11^ using a rational medicinal chemistry approach.^9^ Although the inhibitors have distinct chemical structures, their mechanism of action is similar: selective and potent blockade of the PD-1/L1 interaction, inducing PD-L1 dimerization and internalization, and stimulation-dependent cytokine production.^9^ All three compounds were shown to have equivalent activity to approved mAb-based PD-L1 inhibitors in preclinical studies.

INCB086550, the first compound to be developed, was studied extensively in vitro. INCB086550 reduced tumor growth in CD34^+^ humanized mice and induced T-cell activation gene signatures (eg, *Ifnγ*, *Pdcdc1*, *Lag3*, and *Gzmb*), consistent with PD-L1/PD-1 pathway blockade.^9^ Though chemically distinct, INCB099280 and INCB099318 were expected to perform similarly in vitro. Further, in two triple-negative breast cancer models, a combination of INCB086550 and the Janus kinase TYK2 inhibitor, deucravacitinib, showed significantly enhanced efficacy versus either agent administered alone.^12^

Here, we describe the results of three phase 1 studies that were conducted to evaluate the safety, preliminary efficacy, pharmacokinetics (PK), and pharmacodynamics (PD) of INCB086550, INCB099280, and INCB099318 in patients with advanced solid tumors.

## Methods

### Study designs and patients

Three phase 1 open-label, non-randomized, multicenter, multinational, dose escalation and expansion studies were conducted to evaluate the safety, tolerability, and preliminary efficacy, and to determine the pharmacologically active dose (PAD), maximum tolerated dose (MTD), PK, and PD of each drug in patients with advanced solid tumors: INCB086550—study INCB 86550-102 (study 86550 for short; NCT03762447); INCB099280—study INCB 99280-112 (study 99280 for short; NCT04242199); and INCB099318—study INCB 99318-122 (study 99318 for short; NCT04272034). These studies were conducted over a 6-year period from December 2018 to November 2024.

**Figure 1:**
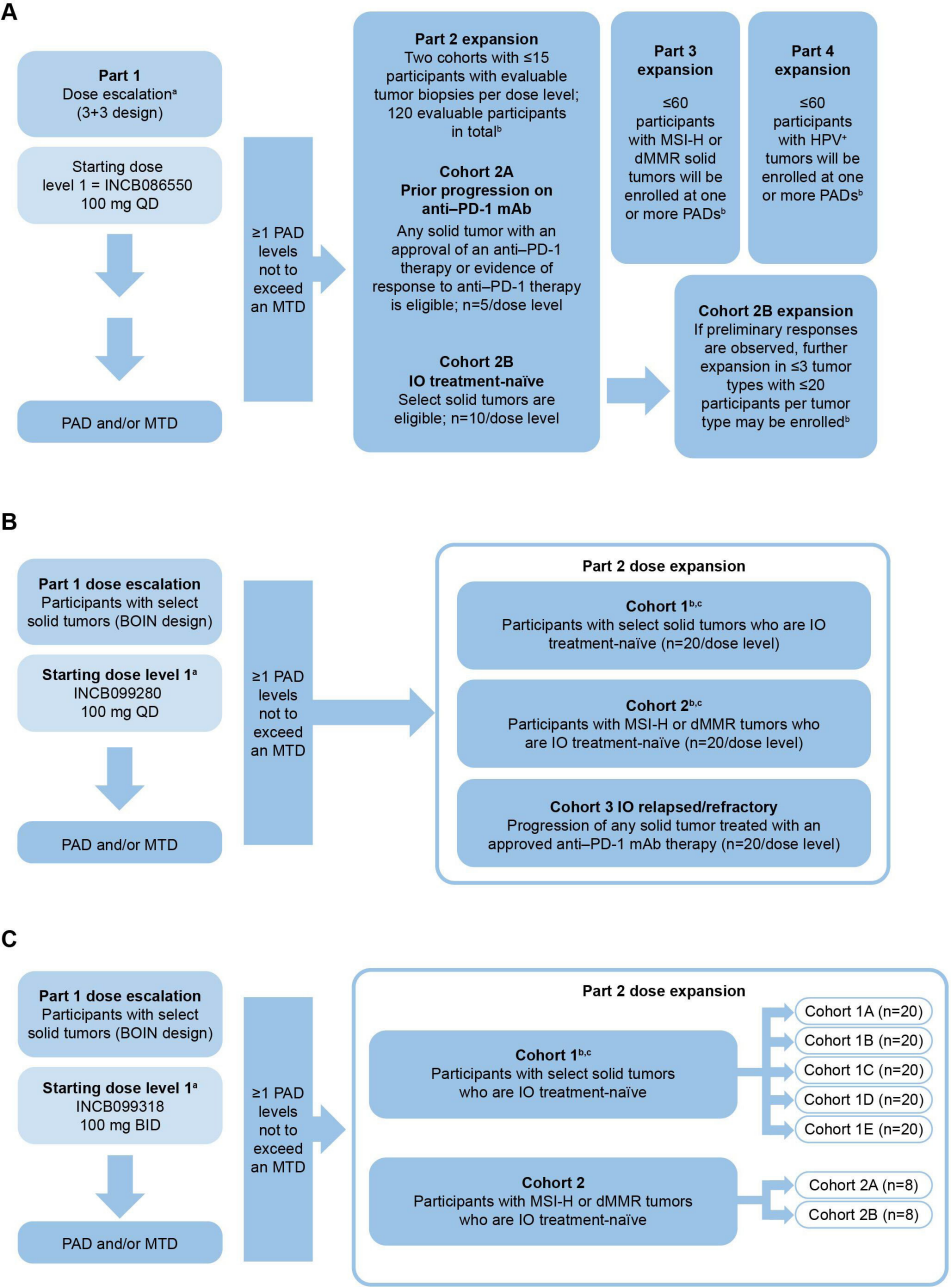
Study designs for phase 1 open-label, non-randomized, multicenter, multinational, dose escalation and expansion studies of (**A**) INCB086550, (**B**) INCB099280, and (**C**) INCB099318 in patients with advanced solid tumors. ^a^Initially, six participants will receive a single dose (100 mg) of INCB086550 on day 3, followed by a timed PK/PD assessment. Dose administration will be initiated on cycle 1 day 1. ^b^9–12 participants who are enrolled in parts 2, 3, or 4 expansion will participate in a food-effect study to evaluate the effect of food on INCB086550 PK. ^a^Dose level may enroll any solid tumor. ^b^A dose level may be expanded in select tumor types based on emerging data per sponsor decision. ^c^Patients must have received locally available standard of care. BOIN, Bayesian optimal interval; dMMR, mismatch repair deficient; HPV, human papilloma virus; IO, immunotherapy; mAb, monoclonal antibody; MSI-H, microsatellite instability-high; MTD, maximum tolerated dose; PAD, pharmacologically active dose; PD, pharmacodynamics; PK, pharmacokinetics; QD, once daily.

Treatment for each of the three agents was limited to 2 years. Study 86550 had a dose escalation part with a modified 3+3 design (part 1) to assess safety and tolerability and identify a PAD and/or MTD, whichever was lower, followed by four expansion cohorts in the expansion phase (part 2): (1) tumors that progressed on prior immuno-oncology (IO) therapies (cohort 2A); (2) select solid tumors naïve to immunotherapy (cohort 2B); (3) microsatellite instability-high (MSI-H)/mismatch repair deficient (dMMR) solid tumors (cohort 3); and (4) human papilloma virus-positive solid tumors (cohort 4) (figure 1A).

Studies 99280 and 99318 each comprised a dose escalation and dose expansion part (figure 1B,C). Dose escalation followed an open-label Bayesian optimal interval design algorithm^13^ with a target toxicity rate of 28% to identify the PAD and/or MTD, with a minimum of three evaluable patients enrolled at each tested dose. The expansion part consisted of three cohorts: (1) patients with select solid tumors who were IO-naïve; (2) a specific patient population with MSI-H or dMMR solid tumors who were IO-naïve; and (3) patients whose disease had progressed on prior treatment with an approved PD-1 mAb (study 99280 only).

Patients in all three studies were adults ≥18 years of age with an Eastern Cooperative Oncology Group performance status score of 0 or 1 who had histologically confirmed advanced solid tumors with measurable lesions (per Response Evaluation Criteria in Solid Tumors (RECIST) v1.1 or Response Assessment in Neuro-Oncology (RANO) for primary brain tumors allowed in study 86550 only) considered non-amenable to surgery or other curative treatments. Patients were required to have disease progression after treatment with available therapies, including any authorized immune checkpoint inhibitors (ICIs) for their tumor type, that were known to confer clinical benefit; or to be intolerant of or ineligible for such treatment. They were also required to have fresh pretreatment and on-treatment biopsies.

Patients who had previously received an anti-PD-L1 therapy or >1 immunotherapy regimen were ineligible for the studies. Other key exclusion criteria were prior receipt of systemic antibiotics within 28 days of the first dose of study treatment due to prior evidence of adverse outcomes with ICI therapy in patients who receive prior antibiotics^14^; presence of a gastrointestinal condition that may have affected drug absorption; active hepatitis B or C, or history of HIV; clinically significant cardiac disease or electrocardiogram (ECG) parameters; and known additional malignancy that was progressing or required active treatment.

Safety committees monitored safety throughout each study and made recommendations to the sponsor’s clinical teams regarding steps to ensure both patient safety and continued ethical integrity of the studies.

### Treatments

Decisions on dose level expansion and determination of the MTD in all studies were based on events observed from the first day of study drug administration through the first 28 days (1 cycle).

The INCB086550 starting dose of 100 mg once daily was based on animal toxicology studies and the estimated coverage of PD-L1 needed for activity/efficacy based on an ex-vivo human whole blood assay. In study 86550, INCB086550 was given in continuous 28-day oral dosing cycles or stepdown/intermittent cycles. Dose escalation was conducted with 28-day cycles from the starting dose up to a maximum of 800 mg two times per day and selected doses were administered in the expansion phase. Stepdown/intermittent cycles, including a 1–2-week dose interruption or dose reduction, were evaluated during dose expansion to optimize benefit versus adverse event (AE) risk.

The starting doses of 100 mg once daily and 100 mg two times per day for INCB099280 and INCB099318, respectively, were based on animal toxicology studies and PK studies in healthy volunteers. Dose escalation was continued in 28-day cycles from the starting dose up to a maximum of 800 mg two times per day, and selected doses were administered in the expansion phase.

### Endpoints and evaluations

The primary endpoints for all three studies were safety and tolerability assessed from the frequency, duration, and severity of AEs; physical and neurological examinations; evaluation of vital signs; ECGs; and clinical laboratory blood and urine sample evaluations. In study 86550, the MTD was identified as the highest dose at which less than one-third of the evaluable patients had a dose-limiting toxicity (DLT) during the first 28 days of treatment. In Studies 99280 and 99318, the MTD was identified as the highest dose at which <28% of evaluable patients had a DLT during the first 28 days of treatment. The PAD was established at a dose that did not exceed the MTD, using PK and PD data.

Treatment efficacy, a secondary endpoint in study 86550 and an exploratory endpoint in Studies 99280 and 99318, was evaluated from the objective response rate (ORR), the disease control rate (DCR), and the DOR. The ORR was defined as the percentage of patients having a complete response (CR) or partial response (PR); the DCR as the percentage of patients having CR, PR, or stable disease (SD) (for ≥12 weeks in Studies 86550, 99280, and 99318); and the DOR as the time from the first documented evidence of CR or PR until the earliest date of disease progression or death due to any cause, if occurring sooner than progression.

AEs were recorded and tabulated by Medical Dictionary for Regulatory Activities preferred term and system organ class, and severity was graded using Common Terminology Criteria for Adverse Events version 5. Potential immune-related AEs (irAEs) were programmatically identified based on a predefined list of preferred terms drawn from prior experience with the PD-L1 inhibitor class.

Tumor imaging was undertaken at screening and every 8 weeks for the first year and every 12 weeks thereafter until progression. Efficacy assessments were by investigator assessment per RECIST v1.1 for all three studies and per RANO for patients with primary brain tumors in study 86550.

Secondary endpoints in all three studies were the single-agent PK parameters determined from plasma samples,^9^ including maximum and minimum concentrations (C_max_ and C_min_), time to C_max_ (t_max_), the area under the concentration-time curve (AUC), and half-life (t_½_). PD assessments were secondary and exploratory endpoints in these studies, consisting of assessments of target engagement, including PD-L1 receptor binding in whole blood and PD-L1 binding on tumor cells. Translational biomarker assessments in Studies 86550, 99280, and 99318, performed using plasma proteomics, were undertaken to demonstrate immune activation and identify prognostic and/or predictive correlative biomarkers for clinical benefit, and included measuring staphylococcal enterotoxin B (SEB)-stimulated secretion of cytokines/chemokines from peripheral blood cells, cytokines, chemokines, and T-cell populations, ctDNA in blood, and PD-L1 expression and immune cells in tumors.^9^

### Statistical analyses

The full analysis set (FAS) included all enrolled patients who received ≥1 dose of the study drug and was analyzed according to the treatment to which patients were initially assigned; the Safety-Evaluable Population included all enrolled patients who received ≥1 dose of the treatment and were analyzed according to the actual treatment received regardless of assigned treatment. The FAS was used to summarize demographics, baseline characteristics, and patient disposition. The Safety-Evaluable Population was used for all safety analyses, except for the DLT analysis, which used the DLT-Evaluable Population (all patients in Part 1 who received ≥75% of planned doses during the DLT observation period or who had a DLT). Patients in the Efficacy-Evaluable (RECIST-evaluable) population were the same as the FAS at the end of the study in all three studies. The PK-Evaluable Population included all patients who received ≥1 dose of study drug and provided ≥1 measurable plasma sample. The Translational Pharmacodynamic-Evaluable Population included all patients who provided a baseline sample, received ≥1 dose of study drug, and provided ≥1 on-treatment sample.

Safety and tolerability data were summarized using descriptive statistics. The ORR and DCR were summarized using descriptive statistics with 95% CIs, and the median DOR with 95% CIs was calculated using the Kaplan-Meier method.

## Results

### Patient disposition

Study 86550 was conducted between December 2018 and November 2023, and enrolled patients from 21 centers across five countries. Study 99280 was conducted between September 2020 and November 2024, and enrolled patients from 16 centers across five countries. Study 99318 was conducted between March 2021 and November 2024, and enrolled patients from 20 centers across seven countries. In study 86550, the FAS comprised 138 patients; the most common reasons for treatment discontinuation were due to disease progression (n=99 (71.7%)) and AEs (n=18 (13.0%)). In study 99280, the FAS comprised 182 patients; the most common reasons for treatment discontinuation were due to disease progression (n=153 (84.1%)) and AEs (n=11 (6.0%)). In study 99318, the FAS comprised 104 patients (some expansion cohorts were not opened to enrollment); the most common reasons for treatment discontinuation were due to progressive disease (n=86 (82.7%)) and AEs and physician decision (both n=4 (3.8%)) (online supplemental table 1). The numbers of patients in each part and dose group for each study are shown in online supplemental table 2.

### Demographics and disease characteristics

Demographics and disease characteristics are shown in table 1. More than half of the patients in each study had received ≥2 prior lines of cancer therapy for advanced or metastatic disease, and 9.6%–16.5% had received prior immunotherapy.

**Table 1:** Demographic and baseline clinical characteristics of patients in studies 86550, 99280, and 99318 (FAS)

Variable	Study		
	86550 (N=138)	99280 (N=182)	99318 (N=104)
Age, median (range), years	65.0 (31.0, 86.0)	63.0 (21.0, 86.0)	58.0 (29.0, 89.0)
Female, n (%)	84 (60.9)	99 (54.4)	62 (59.6)
Race, n (%)			
White	111 (80.4)	82 (45.1)	90 (86.5)
Black	5 (3.6)	5 (2.7)	2 (1.9)
Asian	3 (2.2)	12 (6.6)	4 (3.8)
Other	15 (10.9)	3 (1.6)	2 (1.9)
Not reported* or missing	4 (2.9)	80 (44.0)	6 (5.8)
ECOG Performance Status, n (%)			
0	55 (39.9)	95 (52.2)	38 (36.5)
1	83 (60.1)	87 (47.8)	66 (63.5)
Most common tumor types (≥5%), n (%)			
Anal	15 (10.9)	25 (13.7)	
Breast		12 (6.6)	
Cervical	15 (10.9)	16 (8.8)	18 (17.3)
Colorectal	19 (13.8)	15 (8.2)	6 (5.8)
Esophageal	3 (2.2)	10 (5.5)	4 (3.8)
Gastric	8 (5.8)	6 (3.3)	
Hepatocellular			6 (5.8)
Ovarian			13 (12.5)
MSI high or dMMR, n (%)			
Yes	41 (29.7)	18 (9.9)	1 (1.0)†
Previous lines of cancer therapy in the advanced/metastatic setting, n (%)			
0	15 (10.9)	14 (7.7)	10 (9.6)
1	45 (32.6)	46 (25.3)	23 (22.1)
2	35 (25.4)	45 (24.7)	28 (26.9)
≥3	43 (31.2)	77 (42.3)	43 (41.3)
Prior IO treatment, n (%)			
Yes	15 (10.9)	30 (16.5)	10 (9.6)
No prior cancer therapy	8 (5.8)	7 (3.8)	4 (3.8)
Not reported	0	5 (2.7)	2 (1.9)

* Not reported due to local reporting requirements.

† In study 99318, cohort 2 (n=1) included patients who were MSI high or dMMR only; none were included in the other cohorts in this study.

dMMR, mismatch repair deficient; ECOG, Eastern Cooperative Oncology Group; FAS, full analysis set; IO, immunotherapy; MSI, microsatellite instability.

### Safety

Median durations of exposure were 8.1 weeks (range 0, 106), 8.3 weeks (range 0, 128), and 12.6 weeks (range 0, 104) for INCB086550, INCB099280, and INCB099318, respectively. No patients in studies 86550 and 99318 had a protocol-defined DLT during the DLT observation period. One patient in study 99280 had a DLT with 600 mg INCB099280 once daily (vomiting) and one with 800 mg INCB099280 two times per day (maculopapular rash). There was no MTD identified for any of the three study drugs. The maximum daily administered dose was 1600 mg for all three drugs.

Due to the notable incidence of immune-related neuropathy observed in study 86550, INCB086550 800 mg was established as the maximum total daily dose, and the expansion phase was started at a PAD of 400 mg two times per day, based on integrated assessment of PD, PK, and clinical data. In the absence of an MTD in studies 99280 and 99318, doses were escalated to 800 mg two times per day for INCB099280 and INCB099318.

Treatment-emergent AEs (TEAEs) are summarized in table 2. In study 86550, 136 patients (98.6%) had ≥1 TEAE, 88 (63.8%) had ≥1 treatment-related TEAE, and 19 (13.8%) had ≥1 treatment-related grade ≥3 TEAE. The most common TEAEs were fatigue (n=46 (33.3%)), nausea (n=43 (31.2%)), decreased appetite (n=38 (27.5%)), and vomiting (n=35 (25.4%)). More than half of patients (n=76 (55.1%)) had ≥1 TEAE of grade ≥3; 48 (34.8%) patients had serious AEs (SAEs), and 6 (4.3%) had TEAEs with a fatal outcome, all of which were considered to be unrelated to the study drug (n=1 each for: cerebrovascular accident, intestinal obstruction, lumbar vertebral fracture, hemorrhage intracranial, general physical health deterioration, and dyspnea). Among the 35 (25.4%) patients in study 86550 reporting an irAE, the most frequent were nervous system events (n=14 (10.1%)), which included 10 patients (7.2%) with peripheral sensory neuropathy and two (1.4%) each with immune-mediated neuropathy and peripheral motor neuropathy. In the dose escalation part of the study, one patient experienced peripheral sensory neuropathy of grade ≥3 with INCB086550 400 mg two times per day, and two patients experienced immune-mediated neuropathy of grade ≥3 with INCB086550 800 mg two times per day. To mitigate the risk of nervous system-related irAEs, stepdown/intermittent dosing regimens were explored. However, this did not significantly reduce the risk of immune-related neuropathy.

**Table 2:** Safety summary (safety-evaluable population), TEAEs of grade ≥3 (in ≥2.5% of patients in any group), and irAEs (in ≥1 patient in any group) in studies 86550, 99280, and 99318

Outcome n (%)	Study		
	86550 (N=138)	99280 (N=182)	99318 (N=104)
TEAE	136 (98.6)	176 (96.7)	100 (96.2)
Treatment-related	88 (63.8)	144 (79.1)	69 (66.3)
Treatment-related grade ≥3	19 (13.8)	25 (13.7)	12 (11.5)
SAE	48 (34.8)	52 (28.6)	26 (25.0)
Leading to study drug discontinuation	18 (13.0)	11 (6.0)	4 (3.8)
Leading to study drug interruption	46 (33.3)	49 (26.9)	42 (40.4)
Leading to study drug dose reduction	8 (5.8)	2 (1.1)	4 (3.8)
With fatal outcome*	6 (4.3)	7 (3.8)	4 (3.8)
TEAE grade ≥3	76 (55.1)	69 (37.9)	42 (40.4)
Anemia	12 (8.7)	10 (5.5)	9 (8.7)
Increased AST	6 (4.3)	4 (2.2)	2 (1.9)
Decreased lymphocyte count	5 (3.6)	0	3 (2.9)
Increased ALT	4 (2.9)	5 (2.7)	3 (2.9)
Back pain	4 (2.9)	0	1 (1.0)
Fatigue	4 (2.9)	3 (1.6)	5 (4.8)
Small intestinal obstruction	4 (2.9)	0	1 (1.0)
Hypertension	2 (1.4)	1 (0.5)	4 (3.8)
irAEs (all grades)	35 (25.4)	43 (23.6)	14 (13.5)
Nervous system	14 (10.1)	4 (2.2)	0
Skin reactions	9 (6.5)	11 (6.0)	8 (7.7)
Colitis	4 (2.9)	2 (1.1)	1 (1.0)
Hepatitis	4 (2.9)	11 (6.0)	2 (1.9)
Hypothyroidism	4 (2.9)	5 (2.7)	1 (1.0)
Nephritis	4 (2.9)	3 (1.6)	0
Musculoskeletal and connective tissue	3 (2.2)	1 (0.5)	2 (1.9)
Hyperthyroidism	0	3 (1.6)	0
Pneumonitis	1 (0.7)	3 (1.6)	0

* In study 86550, n=1 each for: cerebrovascular accident, intestinal obstruction, lumbar vertebral fracture, hemorrhage intracranial, general physical health deterioration, and dyspnea. In study 99280, n=1 each for: COVID-19, death (unknown cause), large intestinal obstruction, pneumonia, pulmonary edema, sepsis, and subdural hematoma. In study 99318, n=1 each for: pneumonia, clostridial sepsis, cerebrovascular accident, and streptococcal pneumonia.

ALT, alanine aminotransferase; AST, aspartate aminotransferase; irAE, immune-related adverse event; SAE, serious adverse event; TEAE, treatment-emergent adverse event.

In study 99280, 176 patients (96.7%) had ≥1 TEAE, 144 (79.1%) had ≥1 treatment-related TEAE, and 25 (13.7%) had ≥1 treatment-related grade ≥3 TEAE. The most common TEAEs were asthenia (n=54 (29.7%)), decreased appetite (n=50 (27.5%)), and nausea (n=46 (25.3%)). Overall, 69 (37.9%) patients had ≥1 TEAE grade ≥3 in severity; 52 (28.6%) had SAEs, and 7 (3.8%) patients had TEAEs with a fatal outcome (n=1 each for COVID-19 infection, death (unknown cause), large intestinal obstruction, pneumonia, pulmonary edema, sepsis, and subdural hematoma), all of which were deemed unrelated to the study drug. Among 43 (23.6%) patients reporting an irAE, the most frequently reported were skin reactions and hepatitis, both reported by 11 (6.0%) patients.

In study 99318, 100 patients (96.2%) had ≥1 TEAE, 69 (66.3%) had ≥1 treatment-related TEAE, and 12 (11.5%) had ≥1 treatment-related grade ≥3 TEAE. The most common TEAEs were fatigue (n=34 (32.7%)), constipation (n=25 (24.0%)), and nausea (n=25 (24.0%)). Overall, 42 (40.4%) patients had ≥1 TEAE of grade ≥3 severity; 26 (25.0%) had SAEs, and four (3.8%) had TEAEs with a fatal outcome (n=1 each for pneumonia, clostridial sepsis, cerebrovascular accident, and streptococcal pneumonia), all of which were deemed unrelated to the study drug. Among 14 (13.5%) patients reporting an irAE, the most frequent was skin reactions (n=8 (7.7%)); 5 (4.8%) patients had an irAE of grade ≥3, with hepatitis-related AE being the most common.

### Efficacy

**Figure 2:**
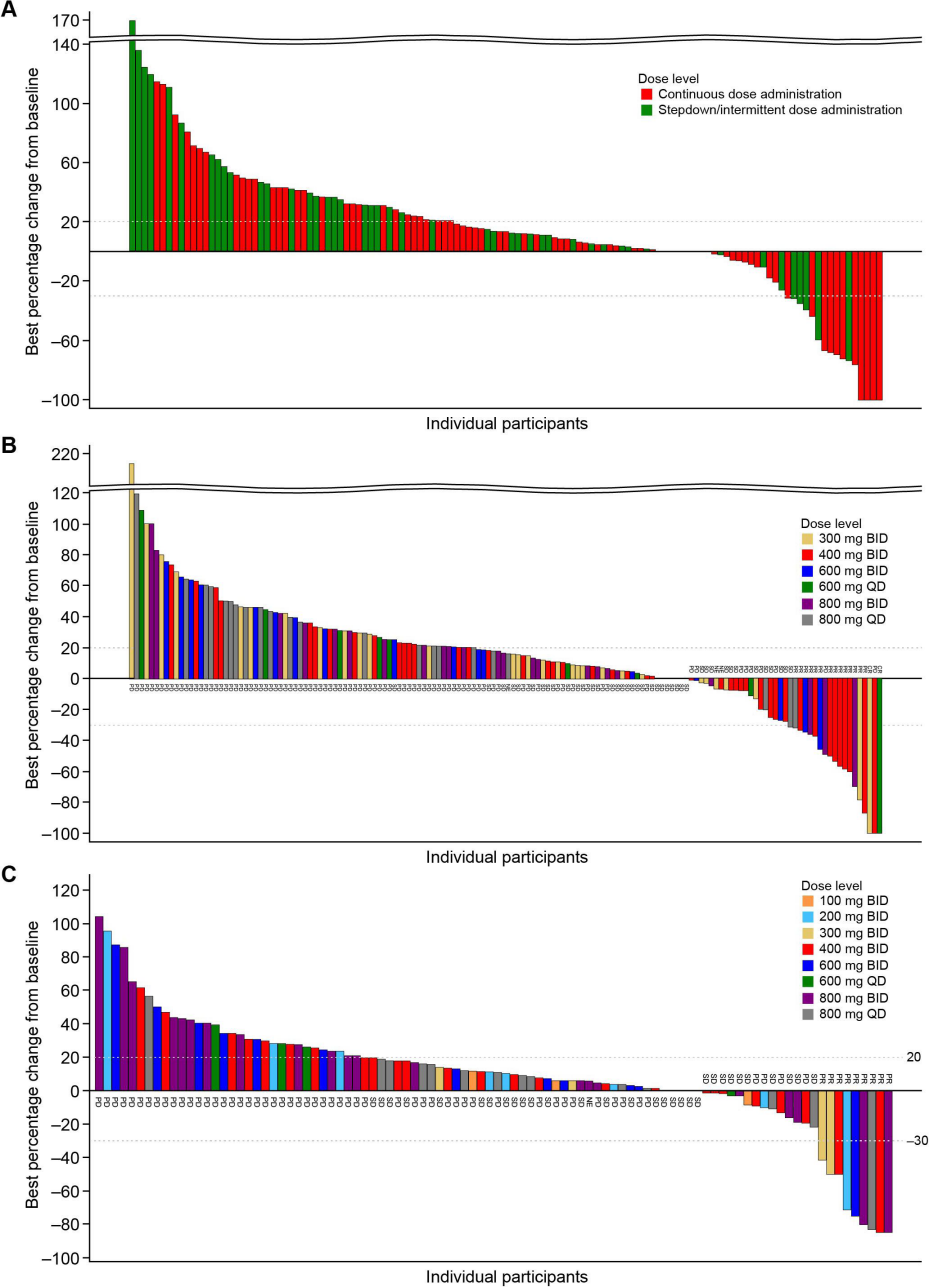
Waterfall plots of best percent change from baseline (efficacy-evaluable population)* in individual patients with advanced solid tumors treated with (**A**) INCB086550, (**B**) INCB099280^†^ and (**C**) INCB099318.^‡^ *Plots include all patients in the Efficacy-Evaluable Population, with baseline and ≥1 valid post-baseline target lesion assessment or measurable enhancing lesion assessment. Best percent change from baseline based on sum of diameters of target lesions by RECIST v1.1 criteria or in sum of product of perpendicular diameters of all measurable enhancing lesions by RANO criteria on each visit. ^†^In study 99280, patients shown were treated with ≥300 mg INCB099280 two times per day and included in the RECIST-evaluable population with baseline plus ≥1 post-baseline target lesion assessment. ^‡^In study 99318, all patients in the RECIST-evaluable population with baseline plus ≥1 post-baseline target lesion assessment are shown. RANO, Response Assessment in Neuro-Oncology; RECIST, Response Evaluation Criteria in Solid Tumors v1.

Waterfall plots of the best percent change from baseline in the sum of diameters of target lesions by RECIST v1.1 criteria, or in the sum of product of diameters of measurable enhancing lesions by RANO criteria (86550 only), are presented for individual patients across each of three studies in figure 2. The efficacy results are summarized in table 3.

**Table 3:** Efficacy outcomes for patients in studies 86550, 99280, and 99318

Outcome	Study		
	86550 N=138	99280 N=182	99318 N=104
Overall			
ORR, % (n) (95% CI*)	10.9 (15) (6.2 to 17.3)	8.8 (16) (5.1 to 13.9)	8.7 (9) (4.0 to 15.8)
DCR, % (n) (95% CI*)	19.6 (27) (13.3 to 27.2)	20.3 (37) (14.7 to 26.9)	21.2 (22) (13.8 to 30.3)
DOR, median, months (95% CI†)	NR (4.2 to NE)	16.8 (1.9 to NE)	21.0 (5.6 to NE)
BOR, n (%)			
CR	3 (2.2)	2 (1.1)	0
PR	12 (8.7)	14 (7.7)	9 (8.7)
SD‡	12 (8.7)	21 (11.5)	13 (12.5)
PD	92 (66.7)	119 (65.4)	72 (69.2)
Not evaluable	8 (5.8)	9 (4.9)	3 (2.9)
Not assessed§	11 (8.0)	17 (9.3)	7 (6.7)

* CIs for ORR and DCR were calculated using the Clopper-Pearson method.

† Kaplan-Meier estimates of median DOR and CIs were calculated using the Kaplan-Meier method.

‡ SDs for ≥12 weeks in studies 86550, 99280, and 99318.

§ Patients in the efficacy-evaluable population who did not have post-baseline overall response assessments by RECIST v1.1 or RANO.

BOR, best overall response; CR, complete response; DCR, disease control rate; DOR, duration of response; NE, not estimable; NR, not reached; ORR, objective response rate; PD, progressive disease; PR, partial response; RANO, Response Assessment in Neuro-Oncology; RECIST, Response Evaluation Criteria in Solid Tumors; SD, stable disease.

The overall ORR across all doses of INCB086550 was 10.9% (15/138; 95% CI 6.2% to 17.3%) and the DCR was 19.6% (27/138; 95% CI 13.3% to 27.2%). Overall, three participants (2.2%) had a CR, and 12 (8.7%) had a PR as BOR. The median DOR for the 15 responders in the study overall was not reached (95% CI 4.2 to not estimable (NE)).

The ORR across all doses for INCB099280 was 8.8% (16/182; 95% CI 5.1% to 13.9%), and the DCR was 20.3% (37/182; 95% CI 14.7% to 26.9%). Two (1.1%) and 14 (7.7%) patients had a BOR of CR and PR, respectively. All responders had received INCB099280 doses ≥300 mg two times per day. The median DOR in the study was 16.8 months (95% CI 1.9 to NE).

For INCB099318, the ORR across all doses was 8.7% (9/104; 95% CI 4.0% to 15.8%), and the DCR was 21.2% (22/104; 95% CI 13.8% to 30.3%); all responders received INCB099318 at total daily doses ≥400 mg. Six of the nine responders were patients with cervical cancer (four with squamous-type histology). No patients had a BOR of CR, and nine (8.7%) patients had a BOR of PR. The median DOR was 21.0 months (95% CI 5.6 to NE).

### Pharmacokinetics

Single oral doses of INCB086550, INCB099280, and INCB099318 were rapidly absorbed (INCB086550: median t_max_ across doses, 1.0–4.0 hours (online supplemental table 3); INCB099280: median t_max_ across doses, ~2 hours (online supplemental table 4); INCB099318: median plasma t_max_, ~1 hour (online supplemental table 5). For INCB086550 and INCB099280, plasma exposure increased approximately in proportion to dose when administered once daily (range: 100–800 mg) and two times per day (range: 200–800 mg). Plasma exposure to INCB099318 (C_max_ and AUC_(0-tau)_) increased approximately in proportion to dose from 100 to 800 mg two times per day. Following repeat dose administrations for once daily regimens, INCB099318 C_max_ increased more than dose proportionally, while AUC_(0-tau)_ increased approximately in proportion with doses from 600 to 800 mg once daily. The accumulation ratios for C_max_ and AUC_0-8h_ ranged from 1.0 to 1.6 and from 1.1 to 1.7, respectively, for the INCB086550 once daily regimens (online supplemental table 6). Greater accumulation was observed with two times per day dosing (accumulation ratios from 2.7 to 4.2 and 3.3 to 5.4 for C_max_ and AUC_0-8h_, respectively). For INCB099280, the accumulation ratios for C_max_ and AUC_0-4h_ ranged from 1.1 to 1.7 and 1.3 to 2.3, respectively, when administered once daily. As was observed for INCB086550, greater accumulation was observed for the two times per day regimens (accumulation ratio range of 2.0 to 4.1 and 1.9 to 6.0 for C_max_ and AUC_0-4h_, respectively). For INCB099318, minimal accumulation was observed following once daily administration: ratios for C_max_ and AUC_0-4h_ ranged from 0.7 to 1.4 and 0.7 to 1.6, respectively. Following two times per day regimens, the ratios ranged from 1.0 to 1.6 and 0.6 to 2.3, respectively. For all three compounds, steady state was achieved by cycle 1 day 8. For INCB099318, overall geometric mean t_½_ values were similar between once daily and two times per day regimens following repeat dosing (3.87 and 3.92 hours, respectively). Given the limited PK sampling time points and duration, t_½_ was not estimated for INCB086550 and INCB099280.

### PD and exploratory biomarker assessments

Samples were collected to evaluate biomarkers of target engagement, PD, response to therapy, and safety. The target engagement assessments for INCB086550 included PD-L1 receptor binding in stimulated whole blood and PD-L1 binding on tumor cells.

INCB086550 demonstrated inhibition of PD-L1 binding in stimulated blood monocytes by flow cytometry, with maximal PD-L1 inhibition consistently occurring between 2 and 3 hours after dosing on cycle 1 day 1 and on cycle 1 day 15. PD-L1 receptor occupancy (RO) in unstimulated whole blood was used to demonstrate target engagement for INCB099280 and INCB099318. Due to the low levels of PD-L1 on the surface of monocytes in many of the samples, the assay was variable. With the exception of the 600 mg once daily dose, the results were dose-dependent for INCB099280. The RO was dose-dependent for INCB099318. For both drugs, the greatest increases in RO were observed at steady-state cycle 1 day 15.

Immune activation and downstream PD activity were seen with INCB086550, INCB099280, and INCB099318. INCB086550 enhanced the production of cytokines and chemokines in whole blood with SEB stimulation in the cohorts tested (200 mg once daily, 200 mg two times per day, and 400 mg two times per day). Increases in the plasma concentrations of soluble target, PD-L1, were observed with both INCB086550 and INCB099280, at 1.4-fold and 1.1-fold, respectively, and were not observed with INCB099318 treatment.

Concentrations of CXCL9, CXCL10, and IFNγ increased after treatment with all three compounds. Additionally, increases in interleukin (IL)-18 and IL-18BP were observed after treatment with INCB099280 and INCB099318, and IL-27 was also increased following treatment with INCB099318. For study 86550, plasma proteomics confirmed the changes observed in sPD-L1, CXCL9, CXCL10, and IFNγ and detected additional markers of immune activation, such as IL-18, IL-27, Granzyme B, and Granzyme H. For study 99280, plasma proteomics confirmed the changes observed and also identified increases in proteins not included in the targeted assessment, including other markers of immune activation such as CD27, CD276 (B7H3), CD8A, GZMA, PD-L2, and HLA-E.

T-cell proliferation, as measured by Ki-67, on CD4 T cells, CD8 T cells, and CD3 T cells, was demonstrated by increases in most patients at doses of ≥200 mg once daily INCB086550, and in most patients treated with INCB099280 or INCB099318.

Taken together, these biomarker changes confirmed target engagement, immune activation, and downstream PD activity with all three compounds. In addition, several biomarkers correlated well with antitumor response.

## Discussion

The PD-1/PD-L1 pathway is a master regulator of tumor immune tolerance and an established therapeutic target in a wide number of malignancies.^15^ Antibody-mediated inhibition of the PD-1 receptor/ligand interaction is effective, but the protracted half-life of these therapies (often lasting several weeks) may unleash unwanted immune-mediated pathology, which can manifest after immunotherapy discontinuation and is sometimes fatal.^16–19^ In addition, repeat intravenous dosing at 2–4 weekly intervals may be a burden to patients’ quality of life and to clinical services through repeated hospital attendance, a finding that led to the development of longer dosing intervals or subcutaneous routes. Thus, development of orally available PD-L1 inhibitors offers an opportunity to further improve the therapeutic index of one of the most successful breakthrough therapies in cancer care.

Phase 1 studies were conducted to evaluate the safety, efficacy, PK, and PD of three orally administered small-molecule PD-L1 inhibitors, INCB086550, INCB099280, INCB099318, in patients with advanced solid tumors. INCB086550 was the first compound evaluated in patients. Because the events of immune-related neuropathy were deemed a significant risk, further clinical development of INCB086550 was discontinued. INCB099280 was the second compound evaluated in phase 1 studies in patients, followed by INCB099318, which was selected for its distinct chemical structure versus INCB086550 and INCB099280.

All three compounds were rapidly absorbed, with the PK being approximately dose proportional. Target engagement and PD activity for the compounds were demonstrated by PD-L1 binding (study 86550), RO (studies 99280 and 99318), and increases in markers of immune activation, including increases in cytokines and chemokines, and increased expression of Ki-67 on CD4 and CD8 T cells following treatment. These results are indicative of the PD-L1/PD-1 interaction,^2 4 20^ as was also shown in preclinical studies.^9^

The safety profiles of the three compounds were generally consistent with mAbs targeting anti-PD-L1, with a few exceptions. All three compounds had lower rates of hypothyroidism than expected, with all-grade incidence ranging from 1% to 3%, as compared with rates ranging from 5% to 8% for nivolumab and pembrolizumab.^21–23^ INCB086550 had a notable rate of peripheral neuropathies (7.2% peripheral sensory neuropathy and 1.4% peripheral motor neuropathy); however, immune-related neuropathy has rarely been observed in patients treated with ICIs alone.^22 24^ Stepdown/intermittent dosing schedules were therefore tested to mitigate this adverse reaction; however, this did not impact rates of peripheral neuropathy.

Neuropathy was rare and not clinically significant in patients treated with INCB099280 or INCB099318, with no patients in study 99318 reporting peripheral sensory neuropathy and only three peripheral neuropathy events (1.6%) reported for study 99280, which were grade 1 (n=2) or grade 2 (n=1) in severity; two of these were considered related to prior chemotherapy.

An investigation into the neuropathy observed with INCB086550 was performed. Immune-mediated AEs are a known side effect of checkpoint inhibition, so assessments were performed to determine if the neuropathy was immune mediated. A review of medical history did not reveal any trends among patients with neuropathy. Increased levels of eosinophils were observed in a subset of these patients along with immune activation in all patients, which was expected with treatment. Analysis of other sequelae, such as stimulation of naturally occurring autoimmunity, paraneoplastic antibodies, antibodies to neuronal components, and HLA typing yielded no observable trends in patients with neuropathy. Thus, no strong evidence to indicate the etiology of the AEs was identified; however, an immune component could not be ruled out.

Preliminary efficacy signals were observed with all three oral PD-L1 inhibitors, with ORRs in the range of 8.7%–10.9%. When responses occurred, they were durable, with median durations exceeding 16 months. The relatively modest response rates are consistent with those observed in the tumor types most frequently represented in these trials, such as cervical, anal, and gastric cancers, for which single-agent PD-(L)1 blockade has shown limited activity.^25–27^ The broad heterogeneity of the enrolled population, including heavily pretreated patients, also likely contributed to the diluted efficacy signal. These findings emphasize that, as with antibody-based PD-(L)1 inhibitors, clinical benefit is likely to depend on careful patient and disease selection supported by predictive biomarkers. Preliminary efficacy results for INCB099280 were promising enough for phase 2 studies to be initiated (NCT05888844, NCT05879822, NCT06337162). However, these studies were terminated due to strategic reasons, and further clinical development of INCB099280 was not pursued. Clinical development of INCB099318 was also not pursued; this was a sponsor decision, not related to safety concerns.

There were some limitations to these studies. First, a comparison of efficacy to historical data with other anti-PD-L1 agents is challenging due to the heterogeneous populations enrolled in the studies and the evolving standard of care in tumors sensitive to immunotherapy. Second, it is unclear why administration of INCB086550 was associated with higher rates and more severe cases of peripheral neuropathy than INCB099280 and INCB099318 or mAbs targeting anti-PD-1/anti-PD-L1. A further limitation was that the reported AUC_0-4h_ was based on data availability and reflected the PK sampling schedule implemented in the three studies; this partial AUC may not capture total systemic exposure. Finally, response stratified by PD-L1 expression was not assessed, as the limited number of responders would have precluded meaningful interpretation.

Although other potential orally administered small-molecule PD-L1 inhibitors have been identified, some of which demonstrate target engagement and immunomodulatory functionality, none have so far progressed beyond clinical trials.^28–31^ The safety profile and level of antitumor activity observed across these phase 1 studies provide a proof of principle that oral targeting of PD-L1 is clinically feasible. This paves the way for future studies to investigate such compounds in selected populations and, importantly, in rational combination regimens with other oral agents, which could broaden the therapeutic impact of PD-L1 inhibition. Furthermore, small-molecule oral PD-L1 therapies have the potential to provide greater convenience and clinical utility compared with intravenous treatment, negating the need for outpatient visits for infusion administration and reducing the risk of infusion reactions.

Overall, clinical development of new immunotherapies remains challenging. There remains an unmet need for treatment of patients who do not respond to anti-PD-L1 therapies in early treatment settings or relapse after an initial response; however, several immunotherapies have had limited success in this space.^32 33^ In order to obtain a better determination of efficacy in solid tumors that are sensitive to anti-PD-L1 therapy, clinical trials in IO-naive patient populations must be conducted. However, feasibility is limited due to the broad availability of anti-PD-L1 therapies across tumor types early in the treatment setting and ethical concerns with giving patients unproven investigational agents.

## Conclusions

Three oral PD-L1 inhibitors were developed and tested in phase 1 studies. All three drugs demonstrated target engagement and immune activation. Although INCB086550 was initially tested in the clinic, the incidence of neuropathy led to the discontinuation of its clinical program. INCB099280 and INCB099318 had acceptable phase 1 safety profiles, and INCB099280 was selected as the lead candidate for phase 2 trials.

## Supplementary material

10.1136/jitc-2026-015334 online supplemental file 1

## Data Availability

Data are available on reasonable request.
