# Supplementary material for: Phase I safety, efficacy, and biomarker response evaluations of three oral PD-L1 inhibitors: INCB086550, INCB099280, and INCB099318
Source: J Immunother Cancer. 2026 Aug 11;14(8):e015334. doi: 10.1136/jitc-2026-015334 (PMC13475261; doi:10.1136/jitc-2026-015334)
Supplement: Supplementary file 1 [file jitc-14-8-s001.docx]

# SUPPLEMENTAL MATERIAL

**Supplemental Table 1** Patient disposition in Studies 86550, 99280, and 99318 (FAS)

|  | Study 86550 | Study 99280 | Study 99318 |
| --- | --- | --- | --- |
| Patients treated (N) | 138 | 182 | 104 |
| Completed treatment, n (%) | 6 (4.3) | 7 (3.8) | 4 (3.8) |
| Discontinued treatment, n (%) | 132 (95.7) | 175 (96.2) | 100 (96.2) |
| Primary reason n (%): |  |  |  |
| Adverse event | 18 (13.0) | 11 (6.0) | 4 (3.8) |
| Progressive disease | 99 (71.7) | 153 (84.1) | 86 (82.7) |
| Death | 4 (2.9) | 4 (2.2) | 3 (2.9) |
| Physician decision | 4 (2.9) | 5 (2.7) | 4 (3.8) |
| Withdrawal by patient | 6 (4.3) | 2 (1.1) | 3 (2.9) |

Completed treatment refers to patients who completed ≥2 years of therapy.

FAS, full analysis set.

**Supplemental Table 2** Numbers of patients (FAS) and dosages in each part and cohort of Studies 86550, 99280, and 99318

| Study 86550 | | | | | | | | | | | | | | | | | | | | | | | | | | | | | | | | | | | | |
| --- | --- | --- | --- | --- | --- | --- | --- | --- | --- | --- | --- | --- | --- | --- | --- | --- | --- | --- | --- | --- | --- | --- | --- | --- | --- | --- | --- | --- | --- | --- | --- | --- | --- | --- | --- | --- |
| Study part / Cohort | 1 | | | 1 | | | 1 | | | 1 | | | 1 | | | 1 | | | 1 | | | 2A | | | 2A | | | 2B | | | 2B | | | 2B | | |
| Dose | 100 mg QD | | | 200 mg QD | | | 200 mg BID | | | 400 mg QD | | | 400 mg BID | | | 800 mg QD | | | 800 mg BID | | | 200 mg BID | | | 400 mg BID | | | 200 mg BID | | | 400 mg BID | | | 400 mg BID 1wk/ off drug 1 wk | | |
| N | 6 | | | 3 | | | 3 | | | 4 | | | 4 | | | 1 | | | 6 | | | 3 | | | 7 | | | 14 | | | 17 | | | 5 | | |
| Study part / Cohort | 2B | | | 2B | | | 3 | | | 3 | | | 3 | | | 3 | | | 3 | | | 4 | | | 4 | | | 4 | | | Total | | |  | | |
| Dose | 400 mg BID 1wk/ 100 mg QD 1 wk | | | 400 mg BID 2 wks/ 100 mg QD 2 wks | | | 200 mg BID | | | 400 mg BID | | | 400 mg BID 1wk/ off drug 1 wk | | | 400 mg BID 1wk/ 100 mg QD 1 wk | | | 400 mg BID 2 wks/ 100 mg QD 2 wks | | | 400 mg BID | | | 400 mg BID 1wk/ off drug 1 wk | | | 400 mg BID 1wk/ 100 mg QD 1 wk | | | — | | |  | | |
| N | 11 | | | 3 | | | 8 | | | 5 | | | 13 | | | 7 | | | 1 | | | 1 | | | 9 | | | 7 | | | 138 | | |  | | |
| Study 99280 | | | | | | | | | | | | | | | | | | | | | | | | | | | | | | | | | | | | |
| Study part / Cohort | 1 | 1 | | | 1 | | | 2/1A | | | 2/1B | | | 2/1C | | | 2/1D | | 2/1E | | 2/2A | | | 2/2B | | | 2/2C | | | 2/3A | | | 2/3B | | | Total |
| Dose | 600 mg QD | 800 mg QD | | | 800 mg BID | | | 300 mg BID | | | 400 mg BID | | | 800 mg QD | | | 600 mg BID | | 800 mg BID | | 300 mg BID | | | 400 mg BID | | | 800 mg BID | | | 400 mg BID | | | 600 mg BID | | | — |
| N | 9 | 10 | | | 14 | | | 20 | | | 20 | | | 14 | | | 13 | | 13 | | 6 | | | 4 | | | 3 | | | 11 | | | 10 | | | 182 |
| Study 99318 | | | | | | | | | | | | | | | | | | | | | | | | | | | | | | | | | | | | |
| Study part / Cohort | 1 | | 1 | | | 1 | | | 1 | | | 1 | | | 1 | | | 1 | | 1 | | | 2/1B | | | 2/1C | | | 2/1D | | | 2/2B | | | Total | |
| Dose | 100 mg BID | | 200 mg BID | | | 300 mg BID | | | 400 mg BID | | | 600 mg QD | | | 600 mg BID | | | 800 mg QD | | 800 mg BID | | | 400 mg BID | | | 800 mg BID | | | 800 mg QD | | | 400 mg BID | | | — | |
| N | 3 | | 8 | | | 5 | | | 10 | | | 4 | | | 14 | | | 10 | | 10 | | | 17 | | | 14 | | | 8 | | | 1 | | | 104 | |

BID, twice daily; FAS, Full Analysis Set; QD, once daily; wk, week.

**Supplemental Table 3** INCB086550 plasma PK parameters after repeat doses: pooled analysis of dose escalation and expansion phases (PK population)

| Dose | N | C_max_ (nM) | t_max_ (h) | C_min_ (nM) | AUC_0-tau_ (h*nM) | AUC_0-8h_ (h*nM) | CL_ss_/F (L/h) |
| --- | --- | --- | --- | --- | --- | --- | --- |
| QD cohorts | | | | | | | |
| 100 mg QD | 6 | 407 ± 205 (370 [50%]) | 3.6 (1.0, 7.5) | 73.0 ± 51.9 (60.7 [72%]) | 4330 ± 2840 (3770 [58%]) | 2060 ± 941 (1900 [45%]) | 42.4 ± 17.9 (38.2 [58%]) |
| 200 mg QD | 3 | 750 ± 309 (699 [52%]) | 2.3 (2.0, 7.5) | 99.3 ± 66.4 (86.8 [68%]) | 7720 ± 3670 (7170 [49%]) | 3970 ± 2560 (3270 [100%]) | 43.1 ± 18.6 (40.2 [49%]) |
| 400 mg QD | 4 | 1480 ± 1260 (1200 [79%]) | 1.6 (1.0, 6.0) | 235 ± 131 (211 [56%]) | 12,500 ± 5180 (11,700 [44%]) | 6200 ± 3240 (5670 [50%]) | 52.6 ± 21.9 (49.2 [44%]) |
| 800 mg QD | 1 | 2290 | 1.7 | 1070 | 34,100 | 14,300 | 33.8 |
| Power function regression analysis of log-transformed data | | | | | | | |
| Exponent on dose (90% CI) | — | 0.859 (0.512, 1.21) | — | 1.10 (0.680, 1.53) | 0.921 (0.601, 1.24) | 0.866 (0.519, 1.21) | — |
| BID cohorts | | | | | | | |
| 200 mg BID | 23 | 2060 ± 1240 (1680 [77%]) | 2.0 (1.0, 5.8) | 991 ± 588 (840 [66%]) | 16,100 ± 9380 (13,600 [69%]) | 11,300 ± 6630 (9470 [71%]) | 25.7 ± 16.9 (21.3 [69%]) |
| 400 mg BID | 27 | 3890 ± 2440 (3200 [76%]) | 2.0 (0.9, 6.0) | 1900 ± 1540 (1330 [139%]) | 28,600 ± 19,200 (23,600 [72%]) | 20,200 ± 12,900 (16,800 [69%]) | 30.0 ± 22.7 (24.5 [72%]) |
| 800 mg BID | 3 | 13,100 ±13,100 (9140 [141%]) | 0.5 (0.0, 1.0) | 7810 ± 8000 (5480 [134%]) | 118,000 ± 120,000 (82,700 [136%]) | 75,500 ± 74,100 (54,200 [130%]) | 18.6 ± 14.4 (13.9 [136%]) |
| Power function regression analysis of log-transformed data | | | | | | | |
| Exponent on dose (90% CI) | — | 1.06 (0.669, 1.45) | — | 0.971 (0.476, 1.47) | 1.03 (0.652, 1.40) | 1.02 (0.655, 1.39) | — |

Study drug concentrations in plasma and urine were assayed at Incyte Corporation (Wilmington, DE, USA) using a validated liquid chromatography/tandem mass spectrometry method, with a linear range of 5 nM (lower limit of quantification) to 2500 nM (upper limit of quantification).

Predose concentrations on cycle 1 day 15 were also used as 12- and 24-hour concentrations in the PK analysis for BID and QD cohorts, respectively. Summary values are presented as mean ± SD (geometric mean [geometric CV%]) except for t_max_, which are presented as median (min, max). Dose proportionality analysis using power mode was performed separately for the BID and QD regimens. Participants enrolled into stepdown/intermittent dose administration cohorts were not included in the pooled analysis across parts.

AUC_0-8h_, area under the concentration-time curve from hour 0 to 8; AUC_0-tau_, area under the steady-state plasma or serum concentration-time curve over 1 dose interval; BID, twice daily; CI, confidence interval; CL_ss_/F, apparent oral dose clearance at steady state; C_max_, maximum concentration; C_min_, minimum concentration at steady state; CV%, percent coefficient of variation; PK, pharmacokinetic; QD, once daily; SD, standard deviation; t_max_, time to maximum concentration.

**Supplemental Table 4** INCB099280 plasma PK parameters after repeat doses: pooled analysis of dose escalation and expansion phases (PK population)

| Dose | N | C_max_ (nM) | t_max_ (h) | AUC_0-tau_ (h*nM) | AUC_0-4_ (h*nM) | C_min_ (nM) | CL_ss_/F (L/h) |
| --- | --- | --- | --- | --- | --- | --- | --- |
| QD cohorts | | | | | | | |
| 100 mg QD | 3 | 172 ± 145 (126 [138%]) | 2.1 (2.0-2.1) | 1410 ± 754 (1250 [74%]) | 421 ± 304 (335 [110%]) | 20.9 ± 3.00 (20.8 [15%]) | 123 ± 86.8 (106 [74%]) |
| 200 mg QD | 3 | 337 ± 429 (178 [247%]) | 2.1 (1.0-4.1) | 2970 ± 3140 (2030 [143%]) | 936 ± 1190 (474 [285%]) | 21.9 ± 5.74 (21.4 [25%]) | 175 ± 134 (130 [143%]) |
| 600 mg QD | 9 | 1410 ± 1740 (610 [299%]) | 2.0 (1.0-4.1) | 17,000 ± 20,900 (8130 [240%]) | 3780 ± 4280 (1750 [290%]) | 326 ± 410 (155 [215%]) | 208 ± 254 (97.2 [240%]) |
| 800 mg QD | 21 | 1820 ± 1580 (1200 [147%]) | 2.1 (2.0-7.2) | 21,200 ± 16,200 (14,400 [140%]) | 5270 ± 4310 (3290 [184%]) | 423 ± 315 (279 [152%]) | 139 ± 220 (73.2 [140%]) |
| Overall | 36 | — | 2.1 (1.0-7.2) | — | — | — | 158 ± 212 (85.0 [151%]) |
| Power function regression analysis of log-transformed data | | | | | | | |
| Exponent on dose (90% CI) | — | 1.150 (0.625, 1.674) | — | 1.235 (0.755, 1.716) | 1.171 (0.616, 1.727) | 1.412 (0.938, 1.886) | — |
| BID cohorts | | | | | | | |
| 200 mg BID | 3 | 303 ± 133 (278 [58%]) | 1.9 (0.0-2.0) | 2870 ± 1470 (2540 [74%]) | 1000 ± 469 (908 [64%]) | 197 ± 114 (167 [90%]) | 121 ± 86.9 (104 [74%]) |
| 300 mg BID | 31 | 1620 ± 891 (1350 [75%]) | 2.0 (0.0-7.0) | 14,900 ± 7800 (12,700 [69%]) | 5440 ± 2820 (4610 [71%]) | 961 ± 558 (804 [72%]) | 38.7 ± 32.9 (31.1 [69%]) |
| 400 mg BID | 40 | 2050 ± 1240 (1730 [66%]) | 2.0 (0.0-7.3) | 19,300 ± 11,800 (16,100 [68%]) | 6980 ± 4240 (5800 [70%]) | 1260 ± 781 (1020 [80%]) | 39.3 ± 25.1 (32.7 [68%]) |
| 600 mg BID | 18 | 2860 ± 1790 (2380 [70%]) | 1.9 (0.0-8.0) | 25,600 ± 16,900 (21,500 [66%]) | 9620 ± 6230 (8030 [68%]) | 1590 ± 1290 (1300 [68%]) | 43.1 ± 24.5 (36.8 [66%]) |
| 800 mg BID | 26 | 3890 ± 2720 (3200 [72%]) | 1.9 (0.0-7.0) | 37,800 ± 28,500 (30,100 [78%]) | 13,300 ± 9890 (10,700 [75%]) | 2480 ± 1980 (1890 [89%]) | 44.3 ± 35.2 (35.0 [78%]) |
| Overall | 118 | — | 2.0 (0.0-8.0) | — | — | — | 42.9 ± 33.7 (34.3 [73%]) |
| Power function regression analysis of log-transformed data | | | | | | | |
| Exponent on dose (90% CI) | — | 1.038 (0.782, 1.295) | — | 1.033 (0.775, 1.291) | 1.031 (0.771, 1.290) | 1.005 (0.726, 1.284) | — |

Predose concentrations on cycle 1 day 15 were also used as 12- and 24-hour concentrations in the PK analysis for BID and QD cohorts, respectively. Summary values are presented as mean ± SD (geometric mean [geometric CV%]) except for t_max_, which are presented as median (min, max).

AUC_0-4h_, area under the concentration-time curve from hour 0 to 4; AUC_0-tau_, area under the steady-state plasma or serum concentration-time curve over 1 dose interval; BID, twice daily; CI, confidence interval; CL_ss_/F, apparent oral dose clearance at steady state; C_max_, maximum concentration; C_min_, minimum concentration at steady state; CV%, percent coefficient of variation; PK, pharmacokinetic; QD, once daily; SD, standard deviation; t_max_, time to maximum concentration.

**Supplemental Table 5** INCB099318 plasma PK parameters after repeat doses: pooled analysis of dose escalation and expansion phases (PK population)

| Dose | N | C_max_ (nM) | t_max_ (h) | AUC_0-tau_ (h*nM) | AUC_0-4_ (h*nM) | C_min, ss_ (nM) | CL_ss_/F (L/h) |
| --- | --- | --- | --- | --- | --- | --- | --- |
| QD cohorts | | | | | | | |
| 600 mg QD | 2 | 2300 ± 962 (2200 [45%]) | 3.1 (2.1-4.0) | 19,700 ± 2010 (19,700 [10%]) | 7190 ± 2630 (6950 [39%]) | 126 ± 67.9 (116 [62%]) | 33.6 ± 3.42 (33.5 [10%]) |
| 800 mg QD | 12 | 5800 ± 4350 (4610 [83%]) | 1.0 (0.7-4.1) | 29,100 ± 15,600 (25,000 [68%]) | 14,000 ± 8770 (11,600 [76%]) | 138 ± 140 (78.9 [167%]) | 42.9 ± 34.4 (35.2 [68%]) |
| Overall | 14 | — | 1.1 (0.7-4.1) | — | — | — | 41.6 ± 31.9 (34.9 [62%]) |
| BID cohorts | | | | | | | |
| 100 mg BID | 3 | 1040 ± 329 (996 [37%]) | 1.0 (1.0-1.1) | 4740 ± 1120 (4660 [23%]) | 2480 ± 1020 (2310 [50%]) | 202 ± 110 (184 [54%]) | 23.9 ± 5.01 (23.5 [23%]) |
| 200 mg BID | 7 | 1910 ± 989 (1740 [48%]) | 1.1 (1.0-2.2) | 6020 ± 2710 (5630 [39%]) | 4100 ± 2170 (3760 [44%]) | 134 ± 72.7 (112 [80%]) | 41.1 ± 13.3 (39.0 [39%]) |
| 300 mg BID | 5 | 2830 ± 1530 (2420 [74%]) | 1.3 (1.0-3.8) | 12,500 ± 3410 (12,000 [33%]) | 6650 ± 2450 (6220 [45%]) | 549 ± 331 (473 [68%]) | 28.6 ± 10.5 (27.4 [33%]) |
| 400 mg BID | 24 | 4180 ± 3350 (3130 [91%]) | 1.2 (0.6-4.1) | 17,800 ± 12,700 (14,200 [77%])^a^ | 10,200 ± 7350 (7990 [82%]) | 529 ± 482 (364 [112%]) | 38.1 ± 25.5 (30.8 [77%])^a^ |
| 600 mg BID | 6 | 6420 ± 3330 (5740 [56%]) | 1.3 (0.0-2.6) | 47,900 ± 31,900 (39,500 [78%]) | 19,400 ± 11,000 (17,000 [61%]) | 2760 ± 2420 (1600 [222%]) | 20.1 ± 12.6 (16.7 [78%]) |
| 800 mg BID | 14 | 7380 ± 2200 (7050 [33%]) | 1.9 (0.5-5.1) | 41,600 ± 20,600 (36,400 [60%]) | 20,300 ± 7100 (19,000 [41%]) | 1700 ± 1550 (1050 [151%]) | 27.8 ± 15.5 (24.1 [60%]) |
| Overall | 59 | — | 1.1 (0.0-5.1) | — | — | — | 32.5 ± 19.9 (27.3 [67%])^b^ |
| Power function regression analysis of log-transformed data | | | | | | | |
| Exponent on dose (90% CI) | — | 0.997 (0.758, 1.237) | — | 1.205 (0.963, 1.448) | 1.117 (0.889, 1.345) | 1.258 (0.853, 1.663) | — |

^a^AUC_0-tau_ and CL_ss_/F derived from n=23.

^b^CL_ss_/F derived from n=58.

N is the number of participants. When there are fewer observations (n) than N, n is presented. Predose concentrations on cycle 1 day 15 were also used as 24-hour concentrations in the PK analysis for QD cohorts. Summary values are presented as mean ± SD (geometric mean [geometric CV%]) except for t_max_, which is presented as median (minimum, maximum). CI for t_max_ is based on Hodges–Lehmann estimator.

AUC0_-4h_, area under the concentration-time curve from hour 0 to 4; AUC_0-tau_, area under the steady-state plasma or serum concentration-time curve over 1 dose interval; BID, twice daily; CI, confidence interval; CL_ss_/F, apparent oral dose clearance at steady state; C_max_, maximum concentration; C_min_, minimum concentration at steady state; CV%, percent coefficient of variation; PK, pharmacokinetic; QD, once daily; SD, standard deviation; t_max_, time to maximum concentration.

**Supplemental Table 6** INCB086550, INCB099280, and INCB099318 plasma accumulation after repeat doses: pooled analysis of dose escalation and expansion phases (PK population)

| **INCB086550** | | | | | | | | | | | | | |
| --- | --- | --- | --- | --- | --- | --- | --- | --- | --- | --- | --- | --- | --- |
|  |  | **C_max_ (nM)** | | | | | | **AUC_0-8h_ (h*nM)** | | | | | |
| Dose | n^a^ | C1 D1 | | C1 D15 | | C1 D15 vs C1 D1^a^ GMR and 90% CI | | C1 D1 | | C1 D15 | | C1 D15 vs C1 D1^a^ GMR and 90% CI | |
| 200 mg BID | 22 | 734 ± 440 (618 [67.5%]) | | 2010 ± 1250 (1640 [77.4%]) | | 2.65 (2.30, 3.05) | | 3280 ± 1580 (2920 [54.1%])^b^ | | 11,300 ± 6360 (9710 [63.9%])^b^ | | 3.32 (2.84, 3.89) | |
| 400 mg BID | 26 | 1380 ± 916 (1130 [72.8%]) | | 3910 ± 2490 (3200 [77.4%]) | | 2.84 (2.37, 3.41) | | 5520 ± 2600 (4940 [52.7%])^b^ | | 22,000 ± 13,700 (18,800 [62.4%])^b^ | | 3.79 (3.32, 4.34) | |
| INCB099280 | | | | | | | | | | | | | |
|  |  | **C_max_ (nM)** | | | | |  | | **AUC_0-8h_ (h*nM)** | | | | |
| Dose | n^b^ | C1 D1 | C1 D15 | | C1 D15 vs C1 D1^a^ GMR and 90% CI | | n^b^ | | C1 D1 | | C1 D15 | | C1 D15 vs C1 D1^a^ GMR and 90% CI |
| 300 mg BID | 11 | 933 ± 531 (781 [75%]) | 1860 ± 997 (1590 [69%]) | | 2.037 (1.265,3.279) | | 7 | | 3020 ± 1040 (2860 [38%]) | | 5800 ± 2530 (5330 [47%]) | | 1.865 (1.260,2.762) |
| 400 mg BID | 18 | 982 ± 945 (622 [142%]) | 2240 ± 1390 (1880 [66%]) | | 3.023 (1.866,4.899) | | 16 | | 2510 ± 2490 (1340 [233%]) | | 7680 ± 4510 (6520 [66%]) | | 4.873 (2.586,9.184) |
| 600 mg BID | 17 | 1620 ± 1320 (1010 [168%]) | 2870 ± 1850 (2360 [72%]) | | 2.344 (1.360,4.042) | | 17 | | 4050 ± 3160 (2320 [268%]) | | 9630 ± 6430 (7950 [70%]) | | 3.430 (1.790,6.570) |
| 800 mg QD | 19 | 2030 ± 1610 (1300 [151%]) | 1970 ± 1580 (1380 [131%]) | | 1.064 (0.599,1.888) | | 15 | | 4600 ± 4000 (2550 [223%]) | | 5560 ± 4600 (3430 [203%]) | | 1.349 (0.599,3.041) |
| 800 mg BID | 25 | 1220 ± 929 (804 [156%]) | 3970 ± 2750 (3260 [73%]) | | 4.056 (2.635,6.244) | | 23 | | 2910 ± 2530 (1750 [173%]) | | 13,100 ± 10,400 (10,400 [78%]) | | 5.950 (3.690,9.593) |
| INCB099318 | | | | | | | | | | | | | |
|  |  | **C_max_ (nM)** | | | | |  | | **AUC_0-8h_ (h*nM)** | | | | |
| Dose | n^b^ | C1 D1 | C1 D15 | | C1 D15 vs C1 D1^a^ GMR and 90% CI | | n^b^ | | C1 D1 | | C1 D15 | | C1 D15 vs C1 D1^a^ GMR and 90% CI |
| 400 mg BID | 15 | 3270 ± 2730 (2330 [110%]) | 4840 ± 3610 (3690 [89%]) | | 1.585 (0.945,2.658) | | 8 | | 4800 ± 3680 (3580 [108%]) | | 9140 ± 6600 (7470 [74%]) | | 2.084 (1.050,4.136) |

Only dose cohorts with ≥10 participants or observations (for ≥1 data point) are included in the table.

^a^ For INCB086550, N is the number of participants. When there are fewer observations (n) than N, n is presented.

^b^ n, number of observations.

Summary values are presented as mean ± SD (geometric mean [geometric CV%]). Participants enrolled into stepdown/intermittent dose administration cohorts were not included in the statistical analysis of accumulation. Only participants with data on both day 1 (day −3 for 100 mg QD cohort) and day 15 were included in the statistical analysis of accumulation.

AUC_0-8h_, area under the concentration-time curve from hour 0 to 8; twice daily; C, cycle; CI, confidence interval; C_max_, maximum concentration; C_min_, minimum concentration at steady state; CV%, percent coefficient of variation; D, day; GMR, geometric mean ratio; PK, pharmacokinetic; QD, once daily; SD, standard deviation.
